# Patient Engagement and Multidisciplinary Involvement Has an Impact on Clinical Guideline Development and Decisions: A Comparison of Two Irritable Bowel Syndrome Guidelines Using the Same Data

**DOI:** 10.1093/jcag/gwy072

**Published:** 2019-01-17

**Authors:** Paul Moayyedi, Megan Marsiglio, Christopher N Andrews, Lesley A Graff, Christina Korownyk, Brent Kvern, Adriana Lazarescu, Louis Liu, Glenda MacQueen, William G Paterson, Sacha Sidani, Stephen J Vanner, Paul Sinclair, Lesley Marshall, Aida Fernandes

**Affiliations:** 1 Farncombe Family Digestive Health Research Institute, McMaster University, Hamilton, Ontario, Canada; 2 IMAGINE Network Patient Research Partner, Hamilton, Ontario, Canada; 3 Cumming School of Medicine, University of Calgary, Calgary, Alberta, Canada; 4 Department of Clinical Health Psychology, Max Rady College of Medicine, Rady Faculty of Health Sciences, University of Manitoba, Winnipeg, Manitoba, Canada; 5 Department of Family Medicine, University of Alberta, Edmonton, Alberta, Canada; 6 University of Manitoba, Winnipeg, Manitoba, Canada; 7 University of Alberta, Edmonton, Alberta, Canada; 8 University Health Network, Toronto, Ontario, Canada; 9 Mathison Centre for Mental Health Research and Education, Department of Psychiatry, University of Calgary, Calgary, Alberta, Canada; 10 GI Diseases Research Unit, Department of Medicine, Queen’s University, Kingston, Ontario, Canada; 11 Centre de Recherche du Centre Hospitalier de l’Universite de Montreal Hopital St-Luc, Division of Gastroenterology, Montreal, Quebec, Canada; 12 GI Diseases Research Unit, Kingston General Hospital, Queen’s University, Kingston, Ontario, Canada; 13 Canadian Association of Gastroenterology, Oakville, Ontario, Canada

**Keywords:** Clinical practice guidelines, IBS, Irritable bowel syndrome, Multidisciplinary team, Patient engagement

## Abstract

**Background and Aim:**

The value of a multidisciplinary group and patient engagement in guideline groups is uncertain. We compared the recommendations of two guidelines that used the same data during the same time frame but with different participants to obtain a “real world” perspective on influence of the composition of guideline groups.

**Methods:**

The Canadian Association of Gastroenterology (CAG) and the American College of Gastroenterology (ACG) recently updated their clinical practice guidelines for the management of Irritable Bowel Syndrome (IBS). Both the CAG and ACG used the same methodology and methodologist and were presented with the same data for interpretation. The ACG group consisted of predominantly academic gastroenterologists, while the CAG group also included general practitioners, a psychiatrist, a psychologist and a patient representative. The CAG group were also asked what components of the group were valuable.

**Results:**

There were 14 statements with the same or similar recommendations. There were 10 statements in the CAG guideline not addressed by the ACG guideline and five recommendations where the opposite was the case. There was one statement that the two groups both addressed, but each group came to different conclusions. CAG members were in 100% agreement that involving a patient and having a multidisciplinary team was valuable and may have played a role in these differing interpretations of the same data in an IBS guideline.

**Conclusions:**

There has been little uptake of patient involvement and multidisciplinary teams in guideline groups. However, this study provides a unique example of added benefit through broader group representation.

Irritable bowel syndrome (IBS) is a common constellation of symptoms that is experienced by 10% to 20% of the population (1). Irritable bowel syndrome imposes a significant burden on the health care system and reduces quality of life (2). In Canada, over 5 million Canadians live with IBS, and $8 billion is attributed to lost productivity each year (3). Given the cost of IBS to the patient and health service, it is important that clinicians are given evidence-based guidance on the optimum management of IBS. They are informed by a systematic review of evidence and follow a transparent methodology to translate best evidence into clinical practice for advancing patient outcomes. There is debate on the ideal composition of a guideline group (4). There is some evidence that a multidisciplinary group (5) and the involvement of patient representatives (6) leads to different questions being asked and some changes in the conclusions reached, but there is a paucity of “real world” data on this topic (7).

Recently, both the Canadian Association of Gastroenterology (CAG) (8) and the American College of Gastroenterology (ACG) (9) updated their clinical practice guidelines for the management of IBS. The ACG Task Force consisted of predominantly academic and clinical gastroenterologists, while the CAG Consensus Group included a broader group of health care professionals including academic gastroenterologists, general practitioners, psychiatrists, and psychologists. Furthermore, the CAG Consensus Group also included a patient representative from the IMAGINE (Inflammation, Microbiome, and Alimentation: Gastro-Intestinal and Neuropsychiatric Effects) Network, a CIHR Strategy for Patient Oriented Research (SPOR) chronic disease network. We compared the two guidelines to see if there was any variation in the statements being evaluated and the conclusions of guidelines that might be attributable to the different compositions of the ACG and CAG groups.

## METHODS

Both and CAG and ACG struck a core group to decide on the scope of the guideline and the statements that would be evaluated. The ACG core group consisted of academic gastroenterologists, but the CAG group also had psychiatry and the patient representative input. Author PM was lead methodologist for both guidelines and conducted a series of systematic reviews on interventions in IBS (10–12) using Cochrane methodology (13) to support the guideline. Both the ACG and the CAG guideline groups were presented with identical data from systematic reviews to inform the statements they were evaluating. Both groups used a modified Delphi approach (14) to reach consensus, and the in-person meetings for each group were held within one week of one other. The lead methodologist (PM) was common to both guideline groups, but all other members were different, and PM kept discussion from each group strictly confidential so neither the ACG nor the CAG group knew of the content of the other group’s discussion. Both the ACG and CAG groups, however, were aware that PM was the methodologist for both guidelines and that the same data were being presented. Both groups used the GRADE approach (15) to evaluate the quality of the evidence and the strength of recommendations. In both cases, the quality of evidence was graded as high to very low by two independent methodologists. There were 12 participants in the CAG guideline group, including six gastroenterologists, two general practitioners, one psychologist, one psychiatrist, one methodologist and one patient representative. The patient representative was a full participant throughout the clinical guidelines development process, contributing to all stages of the guideline according to standard recommendations (16), including development of statements, the prevoting process, the group discussion and voting. The ACG guideline group was comprised of 10 academic and community gastroenterologists.

The scope of the statements was compared between the two guidelines. We also compared the direction of voting and the strength of recommendation made by each group when statements were similar. Finally, we evaluated how the CAG group viewed the multidisciplinary nature of the group and the input of the patient representative using a five-point adjectival scale and the authors were asked the open ended questions for qualitative feedback on the usefulness of the multidisciplinary team and the value of group decision-making.

## RESULTS

Overall, there were 14 statements that were similar between the two groups, with similar or the same recommendations according to GRADE criteria (15) (Table 1). There was one statement in the ACG guideline (9) giving a conditional recommendation for general psychological therapies in IBS. The CAG guideline split this into four separate statements giving conditional recommendation for cognitive behavioural therapy techniques and hypnotherapy but not making a recommendation (either for or against) for psychodynamic psychotherapy or relaxation therapy (8). There were five statements in the ACG guideline not addressed by the CAG guideline and 10 recommendations where the opposite was the case (Table 2). One statement on rifaximin in nonconstipated IBS had differing conclusions between the two groups despite receiving the same data. The ACG guidelines gave this a conditional recommendation for the use of rifaximin in nonconstipated IBS (9), whereas the CAG guideline did not make a recommendation either for or against rifaximin in this patient population (8).

**Table 1.** Similarities between the ACG and CAG IBS guidelines

Category of therapy	CAG guideline	ACG guideline
Diet/fibre	We suggest offering a low FODMAP diet to reduce IBS symptoms	We suggest a low FODMAP diet for overall symptom improvement in IBS patients
	We suggest against a gluten free diet to reduce IBS symptoms	We suggest against a gluten free for overall symptom improvement in IBS patients
	We recommend against wheat bran supplementation to improve IBS symptoms	We recommend psyllium, but not wheat bran, for overall symptom improvement in IBS patients
	We recommend offering psyllium supplementation to improve IBS symptoms	
	We recommend against IBS patients undergo food allergy testing to identify triggers of IBS symptoms	We recommend against exclusion diets based upon antibody or leucocyte activation test for overall symptom improvement in IBS patients
Laxatives	We suggest against offering constipation predominant IBS patients osmotic laxatives in improving OVERALL IBS symptoms	We suggest against PEG for overall symptom improvement in IBS patients
Alternative therapies	We suggest offering IBS patients peppermint oil to improve IBS symptoms	We suggest peppermint oil for overall symptom improvement in IBS patients
Microbiome	We suggest offering IBS patients probiotics to improve IBS symptoms	We suggest probiotics, taken as a group, to improve global symptoms as well as bloating and flatulence in IBS patients
Antidepressants/ Psychological therapies	We recommend offering IBS patients low dose tricyclic antidepressants to improve IBS symptoms	We recommend tricyclic antidepressants for overall symptom improvement in IBS patients
	We suggest offering IBS patients SSRIs to improve IBS symptoms	We suggest SSRIs for overall symptom improvement in IBS patients
	We suggest offering IBS patients cognitive behavioral therapy to improve IBS symptoms	We suggest some psychological therapies (provider-directed cognitive behavioural therapy, relaxation therapy, hypnotherapy, and multi-component psychological therapy) for overall symptom improvement in IBS patients
	We suggest offering IBS patients hypnotherapy to improve IBS symptoms	
	The consensus group does not make a recommendation (neither for or against) offering IBS patients relaxation techniques to improve IBS symptoms	
	The consensus group does not make a recommendation (neither for or against) offering IBS patients short-term psychodynamic psychotherapy to improve IBS symptoms	
Pharmacological therapies	We suggest offering IBS patients certain antispasmodics (dicyclomine, hyoscine, pinaverium) to improve IBS symptoms	We suggest certain antispasmodics (otolinium, hyoscine, pinaverium, cimetropium, drotaverine and dicyclomine) for overall symptom improvement in IBS patients
	We recommend offering constipation-predominant IBS patient linaclotide to improve IBS symptoms	We recommend linaclotide for overall symptom improvement in IBS-C patients
	We suggest offering constipation-predominant IBS patient lubiprostone to improve IBS symptoms	We recommend lubiprostone for overall symptom improvement in IBS-C patients
	We suggest offering diarrhea-predominant IBS patient eluxadoline to improve IBS symptoms	We suggest eluxadoline for overall symptom improvement in IBS-D patients
	We suggest against offering diarrhea-predominant IBS patient continuous loperamide use to improve IBS symptoms	We suggest against loperamide for overall symptom improvement in IBS patients

**Table 2.** Differences between the ACG and CAG IBS guidelines

Category of therapy	CAG guideline	ACG guideline
Alternative therapies	We suggest against offering herbal remedies to IBS patients to improve IBS symptoms	Not addressed
	We suggest against offering acupuncture to IBS patients to improve IBS symptoms	Not addressed
Microbiome	Not addressed	We suggest against the use of prebiotics and synbiotics for overall symptom improvement in IBS patients
	The consensus group does not make a recommendation (neither for or against) offering diarrhea predominant IBS patients one course of rifaximin to improve IBS symptoms	We suggest the non-absorbable antibiotic rifaximin for reduction in global IBS symptoms as well as bloating in non-constipated IBS patients
Pharmacological therapies	We suggest against offering diarrhea predominant IBS patients cholestyramine in improving IBS symptoms	Not addressed
	We suggest against offering constipation predominant IBS patients prucalopride in improving OVERALL IBS symptoms	Not addressed
	Not addressed	We suggest plecanatide for overall symptom improvement in IBS-C patients
	Not addressed	We suggest alosetron for overall symptom improvement in female IBS-D patients
	Not addressed	We suggest against 5-aminosalicylates (5-ASAs) for overall symptom improvement in IBS patients
Exercise	Not addressed	We suggest against the use of exercise for overall symptom improvement in IBS patients
Diagnostic testing	We suggest IBS patients have serological testing to exclude celiac disease	Not addressed
	We recommend against testing for CRP in IBS patients to exclude inflammatory disorders	Not addressed
	We recommend against routine testing for fecal calprotectin in IBS patients to exclude inflammatory disorders	Not addressed
	We recommend against IBS patients < 50 years of age without alarm features ROUTINELY having colonoscopy to exclude alternate diagnoses	Not addressed
	We recommend against IBS patients < 50 years of age with alarm features ROUTINELY having colonoscopy to exclude alternate diagnoses	Not addressed
	We recommend IBS patients ≥ 50 years of age have a colonoscopy to exclude alternate diagnoses	Not addressed

**Figure 1.**
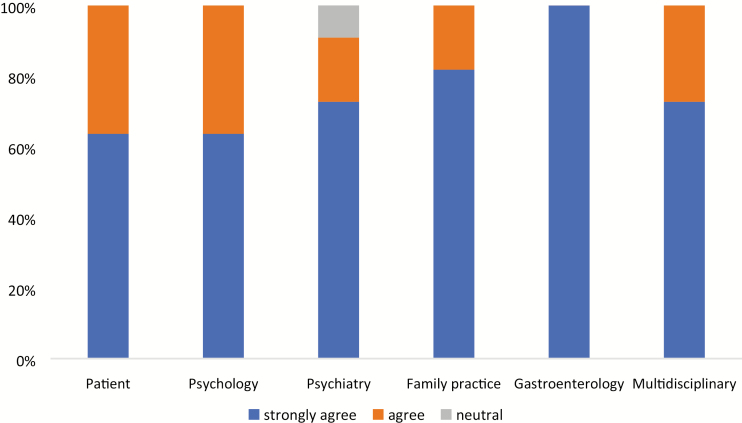
Opinion of the CAG guideline consensus group on the value of each discipline involved in the IBS guideline

The survey conducted with the CAG Consensus Group members on the guideline development process was completed by 10 of 11 of participants (PM did not vote as the methodologist common to both guideline groups). All participants agreed or strongly agreed that the multidisciplinary nature of the group helped form their opinion (Figure 1). The descriptive words used to respond to the open-ended questions are outlined in Box 1. The most commonly used words, spontaneously provided by at least 25% of the participants were ‘professionals’, ‘experience’, ‘primary care’, ‘helpful’, ‘patient preference’ and ‘patient-centric;. According to one of the CAG consensus group members, “the broad spectrum of clinical practice settings and expertise made the consensus meeting a very fruitful one. We all see different aspects of the same disease, therefore having a broader consensus group and patient involved in the process generated a much fuller and richer picture.” The patient representative commented that involving the lived experience perspective and wide ranging clinical viewpoints can “be assuring to end users and adds greater credibility to the guideline document.”

**Box 1. Words used to describe the experience of working in the CAG guideline group**

**Patient-centric** Wide range

**Primary care** Relevant

**Helpful** Patient preference

**Expertise** Values

**Professionals** Practical

**Experience** Assessment

Comprehensive Generalist

Credibility Sound

Various viewpoints Specialist

Varied experts Spectrum

Complex Experts

Multi-sided Real-world

Asset Different voices

Valuable

## DISCUSSION

We present one of the few examples in the literature of results of a “real world” setting where two guideline groups were given the same data and same methodology for evaluating the quality of the data and reaching consensus. Both groups contained an optimum number of participants, usually regarded as between six and 12 members (17). Many of the statements and decisions were very similar, but there were key differences, suggesting that multidisciplinary groups and engaging patients is important. The Institute of Medicine in the US recommended in 2011 that guidelines should have greater patient representation in developing priorities and being involved in the process. Despite this, of the 101 US organizations reviewed, less than 10% had a requirement that patients be involved in guidelines groups, according to their published methodology for conducting guidelines (18) five years after these recommendations were published. A greater proportion ask for patient organization input once the guideline is written, but this is an inadequate approach to ensuring that the patient voice is heard because they can only react to what is written in front of them rather than be involved in process from conception to completion (16). This is particularly important because one component of the GRADE approach (15) is to capture patients’ values and preferences in making recommendations. This clearly cannot be done adequately if a patient is not involved in the process. The reasons for this slow uptake are multifactorial but may relate to the lack of evidence for the value of this approach. A systematic review of patient engagement in guidelines (19) identified 71 articles that reported on the value of patient involvement in the guideline process. Most were qualitative and focused on how engaging patients improves the incorporation of values and preferences in the guideline (19). None had a comparison guideline where patient engagement was not used and the same data were evaluated at approximately the same time. Our study is a rare example of this type of comparison and suggests patient engagement can add value. This is particularly true of the rifaximin statement where no recommendation was made in the CAG guideline, but the ACG gave a conditional recommendation for this drug in nonconstipated IBS. Both groups struggled with the modest efficacy of the drug, the expense of the product and concerns around antibiotic resistance. The patient (MM) involved in the CAG guideline pointed out in the in-person meeting that patients view antibiotics as being used to treat infection, and the clinician would be signalling to their IBS patient that they had an ‘infectious disease’, which does not fit with the paradigm that they have for their problem. This concern resonated with the general practitioners in the group and some of the other panel members, as well. It is also supported by research that suggests patients need a consistent paradigm for why they have their IBS symptoms, and if there is no clear message on what is causing their disease and treatments that fit within that paradigm, then patient satisfaction is less and outcomes could be poorer (20). These issues were not raised in the ACG group, and this may be one reason why a different conclusion was reached. The challenge in interpreting this qualitative information is that this is just one example, and it is difficult to obtain quantitative comparative information from a large number of guidelines to provide robust evidence. Nevertheless, our observation is consistent with another study that also found patient engagement in a guideline on amyloid positron emission tomography in dementia led to different recommendations from evaluating the same data (21). This study also found that most recommendations were very similar, but there was value in adding the patient perspective. On occasion, this did lead to different recommendations, which were seen as more patient-centric. This is the only other example in the literature that we could identify, but it is reassuring that this gives a similar message.

Differences in the two IBS guidelines do not just relate to patient involvement. Research suggests that whether a guideline group is homogeneous or heterogeneous depends on the disease being studied (7). When the disease being evaluated is highly specialized with little impact on other disciplines, then a guideline group consisting of specialists (and a patient representative) may well be appropriate. However, IBS is a disease primarily managed in primary care and with a strong overlap of psychological comorbidity, where psychological interventions have been shown to be effective. It is therefore logical to include these groups in the guideline panel; they were also important in shaping the guideline. As stated previously, the involvement of primary care was an important factor in the decision regarding rifaximin. The CAG guideline also divided psychological interventions into four categories, while the ACG guideline considered them as all one intervention. The CAG position on psychological interventions is therefore more nuanced, emphasizing that data are more robust for some psychological interventions compared with others.

There are some limitations of this work. Any research in this area has the challenge that it is difficult to know with certainty what the ‘correct’ recommendations should be. This work shows that, although many recommendations are the same, there are some important differences. It does not say which guideline is the best reflection of the evidence of which should be followed by clinicians. This is only one disease and two groups, and different results may be obtained in different diseases and with different settings. Finally, not all differences between the guidelines are attributable to differences in group composition. For example, the Canadian guideline evaluated prucalopride, which is not available in the United States, while the opposite is true of alosetron. Thus, local availability of interventions is likely to be the explanation for some of the differences observed.

In conclusion, we present a ‘real world’ example of the same data being evaluated by two guideline groups: one consisting of a single specialty and the other being multidisciplinary with patient involvement. Differences between the two guidelines emphasizes that the composition of the guideline group needs to be chosen carefully and should represent all types of health care professions working in the disease of interest and should include a patient with the disease so that this valuable perspective can be captured.
